# Simultaneous dislocation of the radial head and distal radio-ulnar joint without fracture in an adult patient: a case report and review of literature

**DOI:** 10.1186/s12893-020-00717-8

**Published:** 2020-04-15

**Authors:** Xiang-Yun Jin, Wen-Bo Zhao, Yu-Qi Dong, Yi-Gang Huang

**Affiliations:** 1 Department of Orthopedic Trauma, Renji Hospital, School of Medicine, Shanghai Jiao Tong University, Shanghai, 200127 People’s Republic of China; 2 Department of Orthopedic Surgery, Shanghai Jiao Tong University Affiliated Sixth People’s Hospital, 200233 Shanghai, People’s Republic of China

**Keywords:** Forearm injuries, Soft tissue injuries, Joint instability, Case report

## Abstract

**Background:**

Simultaneous dislocation of the radial head and distal radio-ulnar joint without fracture (Criss-Cross Injury) in an adult patient is rarely reported in previous studies. The pathological changes and injury patterns have not been clearly demonstrated.

**Case presentation:**

A 26-year-old woman presented with acute pain of the right wrist and elbow after a fall from cycling. Physical examination revealed an unstable elbow and wrist joint. Plain radiographs showed volar dislocation of the radial head and dorsal dislocation of the distal radius without associated fracture, forming a criss-cross appearance of the ulna and radius on the lateral radiograph. MRI images confirmed partial rupture of the proximal interosseous membrane from its dorsal attachment on the radius, as well as partial rupture of the medial collateral ligament. Conservative treatment failed because the radiocapitellar joint and distal radio-ulnar joint could not be simultaneously reduced. Surgical exploration revealed a highly unstable radial head, but the annular ligament was found to be intact. Manual force was applied to reduce the radial head and a percutaneous K-wire was used to stabilize the proximal radioulnar joint with the forearm in full supination. After surgery, the elbow was immobilized in 90° flexion by a long arm cast for 4 weeks. The K-wire was removed at 6 weeks postoperatively. At 18 months postoperatively, the patient had regained a full range of flexion and extension, with normal supination and a slight limitation in pronation.

**Conclusions:**

The proximal IOM, especially the dorsal band, was injured in Criss-Cross injuries, while the central part of the IOM remained intact. This injury pattern distinguished itself from Essex-Lopresti injury, which mainly involves rupture of the central band of the IOM.

## Background

Simultaneous dislocation of the radial head and distal radio-ulnar joint without fracture in an adult patient is rarely reported in previous studies with only 7 documented cases [1–7]. The typical radiographic changes involve volar dislocation of the radial head and dorsal dislocation of the distal radius, forming a criss-cross appearance of the ulna and radius on the lateral radiograph [1]. Unlike Essex-Lopresti injuries, the central band of the interosseous membrane (IOM) was believed to be intact with the proximal and distal IOM being simultaneously injured. However, the pathological changes and injury patterns were unclear and speculated only from radiographs and personal experiences. No exact proof has been provided in previous studies. In this case, additional MRI images and surgical findings provided clues to the pathological changes and injury patterns in this specific injury.

## Case presentation

A 26-year-old woman presented to the emergency department immediately after a bicycle accident in which she fell on the ground with an outstretched hand. She complained of pain at her right wrist and elbow, especially when she rotated the forearm. On physical examination, the forearm was held in supination with the elbow in 90° flexion and the wrist in the neutral position. A superficial abrasion was first noted on the palmar surface of the right wrist. Tenderness was triggered with direct palpation on the distal radio-ulnar joint (DRUJ), as well as the lateral and medial side of the elbow. No tenderness was detected with palpation on the middle portion of the forearm. The active range of motion of elbow joint was flexion 110°/extension 180°, and rotation of forearm was supination 80°/pronation 40°. She had a normal movement of the fingers and intact sensation in the right hand.

**Fig. 1:**
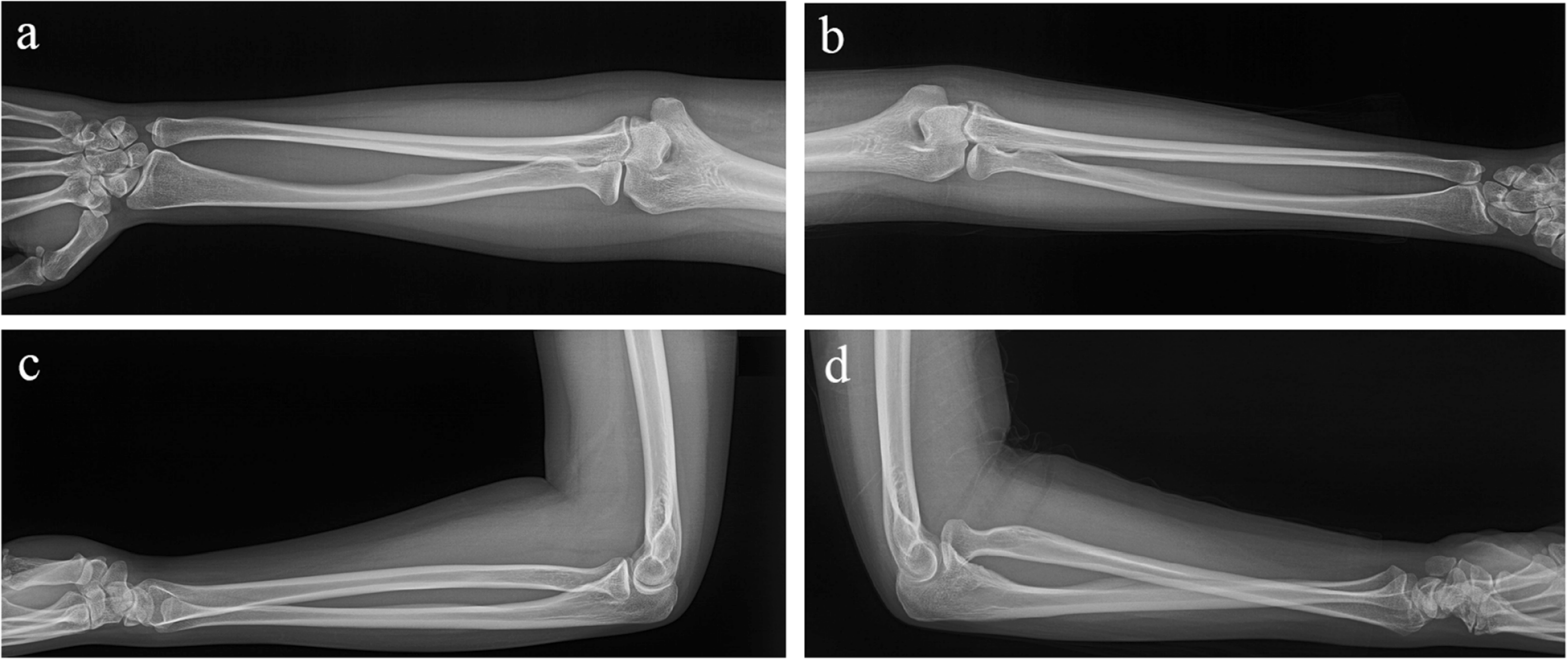
Radiographs of the bilateral forearm. **a**: Anteroposterior view of the normal side; **b**: Anteroposterior view of the injured side; **c**: Lateral view of the normal side; **d**: Lateral view of the injured side demonstrating volar dislocation of the radial head and dorsal dislocation of the distal radius without associated fracture, thus forming a criss-cross appearance on the lateral radiograph

**Fig. 2:**
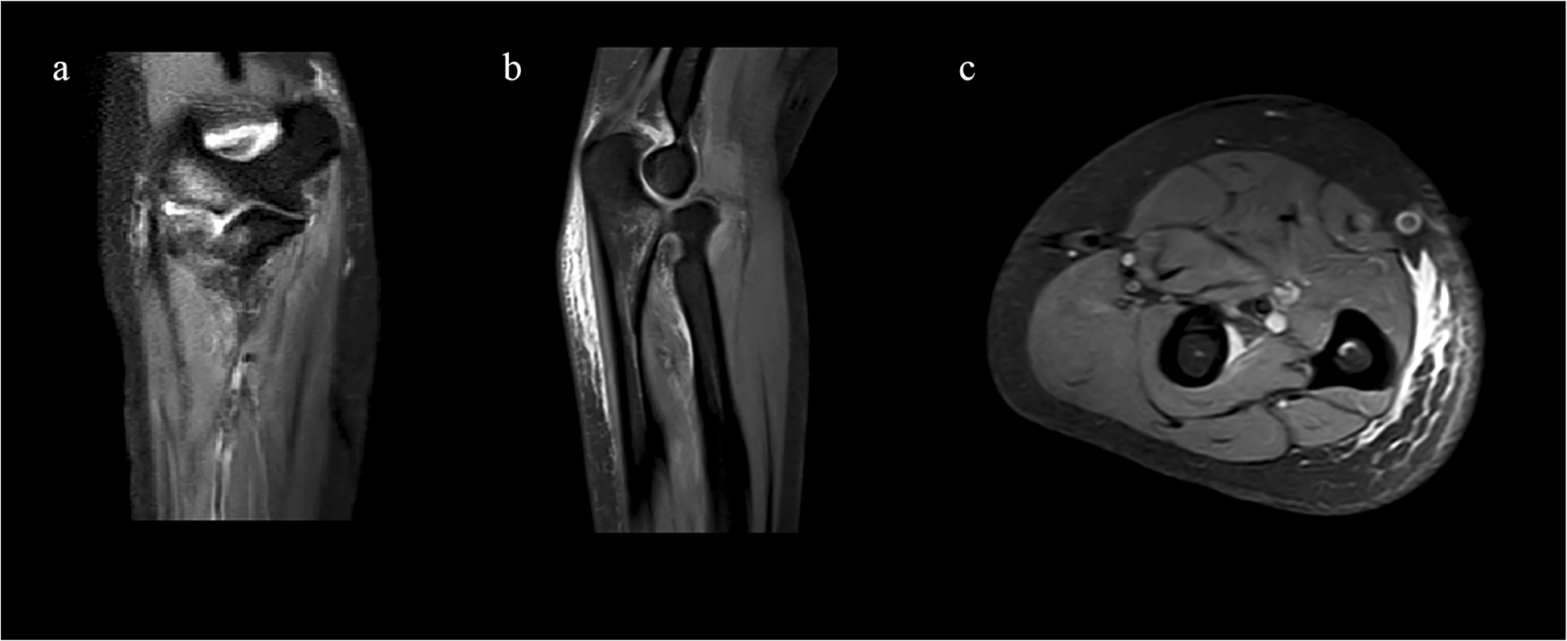
Preoperative MRI images of the right forearm (MRI Sequence: PD_TSE_SPAIR). **a**: Coronal image shows bone contusion of the capitellum of the humerus and proximal ulna (White arrow), as well as partial rupture of the medial collateral ligament (Yellow arrow). **b**: Sagittal image shows high signal around the proximal IOM (Red arrow). **c**: Axial image shows rupture of the proximal IOM from the dorsal attachment (Red arrow). R: Radius; U: Ulna

Radiographs of both forearms were taken for comparison, revealing volar dislocation of the radial head and dorsal dislocation of the distal radius without associated fracture on the injured side, which formed a criss-cross appearance on the lateral radiograph (Fig. 1). Computed Tomography (CT) scan was also applied to rule out associated fractures. To further evaluate the soft tissue injuries, Magnetic Resonance Imaging (MRI) of the forearm was taken, revealing partial rupture of the proximal IOM from its dorsal attachment and no impairment of the middle portion of the IOM, as well as partial rupture of the medial collateral ligament (MCL) (Fig. 2). Aside from ligamentous injuries, bone contusion was also detected on the capitellum of the humerus and the proximal ulna on MRI images.

**Fig. 3:**
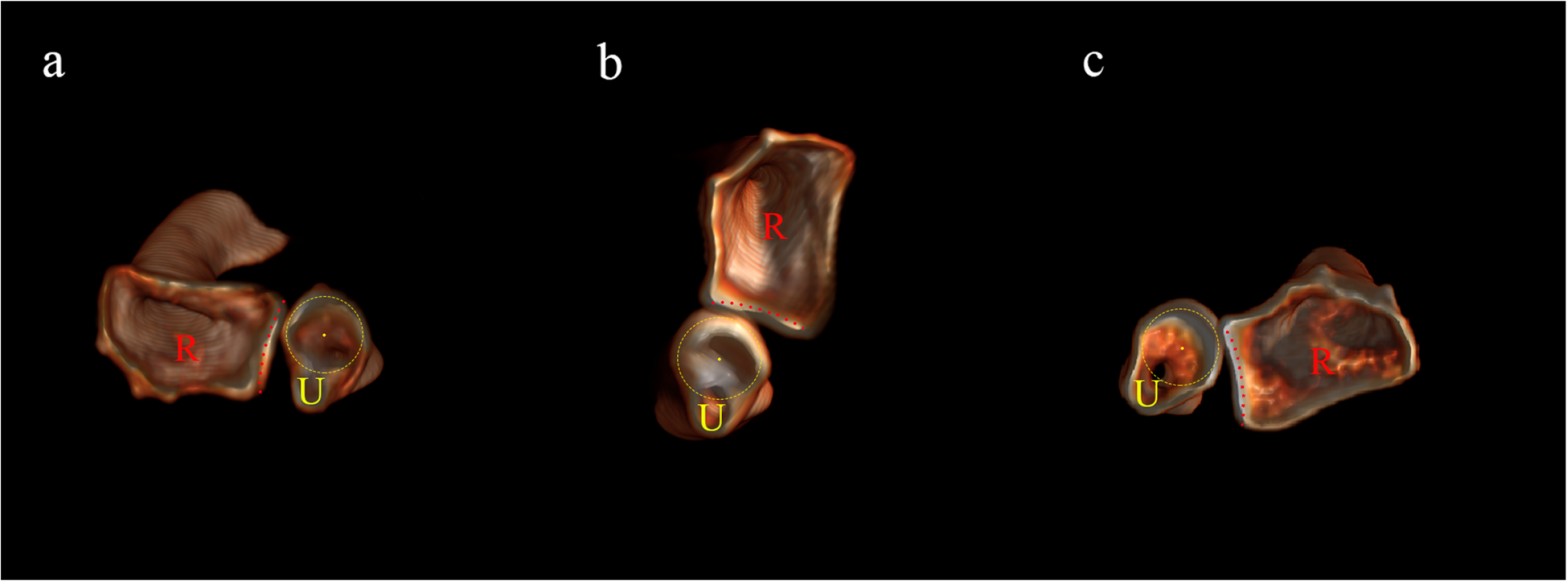
Three dimensional CT reconstruction of the DRUJ with the forearm in different rotation position. **a**: The distal radius dislocated dorsally with the forearm in supination. **b**: The DRUJ was reduced with the forearm in neutral position. **c**: The distal radius dislocated palmarly with the forearm in pronation. Red dotted line: Sigmoid notch; Yellow dotted circle: Ulnar head; R: Radius; U: Ulna

The patient was first diagnosed as “Simultaneous dislocation of the radial head and distal radio-ulnar joint without fracture”. We inclined not to diagnose this injury as an Essex-Lopresti injury because the central part of IOM seemed intact. Conservative treatment was firstly tried with closed reduction in the emergency room without anesthesia. With the elbow in 90° flexion, the forearm was successively immobilized in either supination, pronation or neutral rotation with a long-arm cast. In each position, CT scan of the whole forearm was taken (Fig. 3). However, the radiocapitellar joint and the DRUJ could not be simultaneously reduced in either position.

The patient was then introduced to an expert in elbow surgery and advised to undergo surgical exploration. Before the surgery started, the stability of the elbow and the DRUJ were respectively tested under general anesthesia. During the Valgus Elbow Stress Test, the laxity of the elbow was increased and the radial head seemed to shift forward under a valgus force, indicating that the medial collateral ligament of the elbow and the annular ligament might be injured. The Ballottement Test also showed a positive result, revealing an unstable DRUJ.

**Fig. 4:**
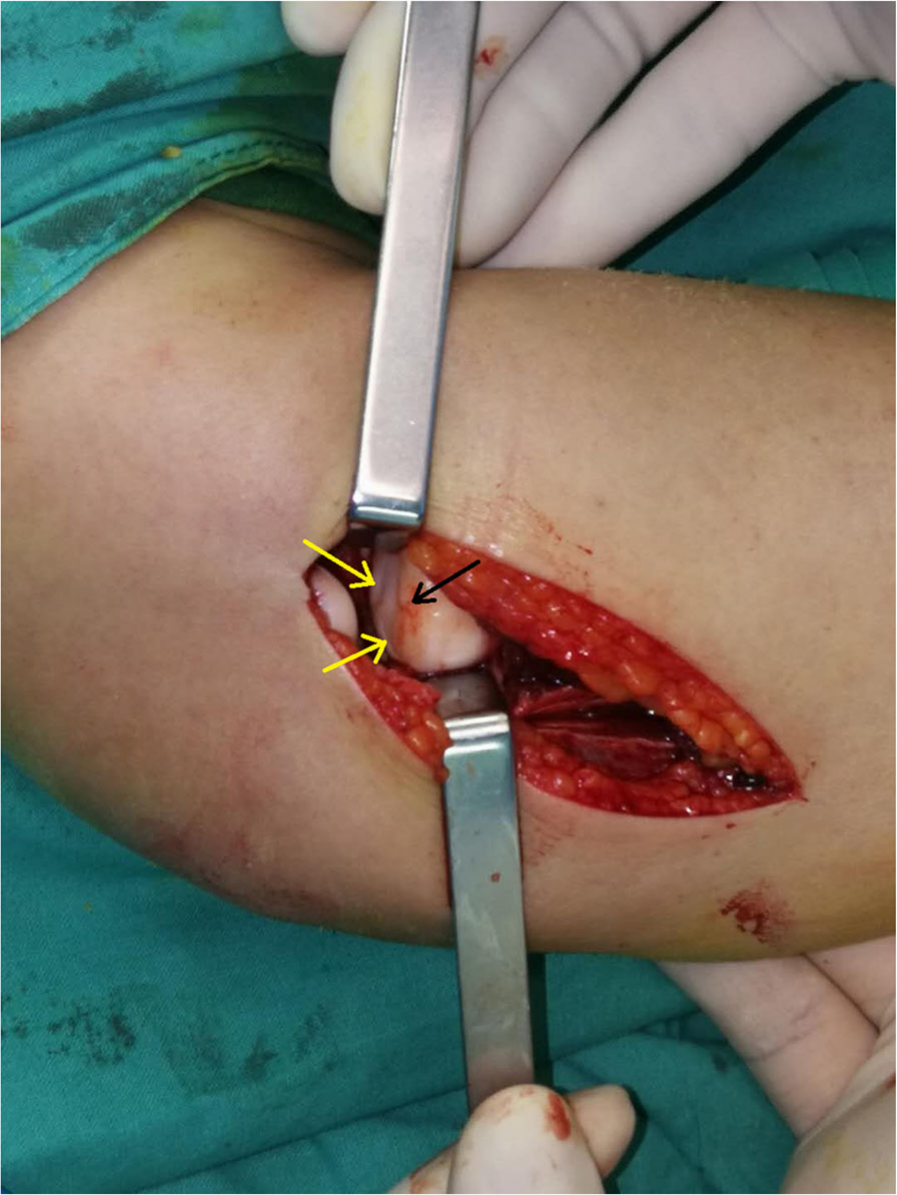
Operative exploration revealed an abnormal radial head. Black arrow: Convex radial head. Yellow arrow: Bumpy articulation

**Fig. 5:**
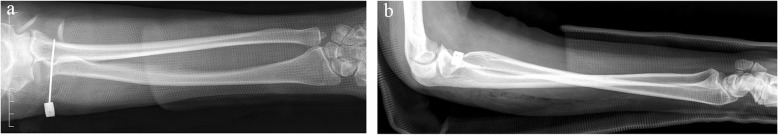
Postoperative radiographs of the injured forearm. **a**: Anteroposterior view; **b**: Lateral view

After the examination, the surgery was performed, aiming to explore the lateral side of the elbow and repair the potentially injured annular ligament. A lateral approach of the elbow was performed. After dividing the subcutaneous tissues, the lateral collateral ligament revealed itself to be intact. Then, a longitudinal incision was made on the lateral collateral ligament to expose the annular ligament and the humeroradial joint. Surprisingly, the annular ligament was found to be perfectly intact, while the radial head was in a slightly anteriorly dislocated position. Further inspection of the radial head revealed an abnormal, convex and bumpy articular surface (Fig. 4). An intraoperative Valgus Elbow Stress Test showed that the radial head would dislocate anteriorly under a valgus force. To reduce the radial head, rotation of the forearm was tried first and the results were not satisfying. So we apply a manual force upon the anterior part of the radial head to achieve a well-aligned reduction while the forearm was in full supination and the elbow was in 90° flexion. Subsequently, a 2.0-mm percutaneous K-wire was used to stabilize the proximal radio-ulnar joint (PRUJ). Intraoperative radiographs showed that the radial head and the DRUJ were simultaneously reduced (Fig. 5). After closing the wound, a long arm cast was used to keep the forearm in supination, 90° of elbow flexion, and the wrist in neutral deviation.

**Fig. 6:**
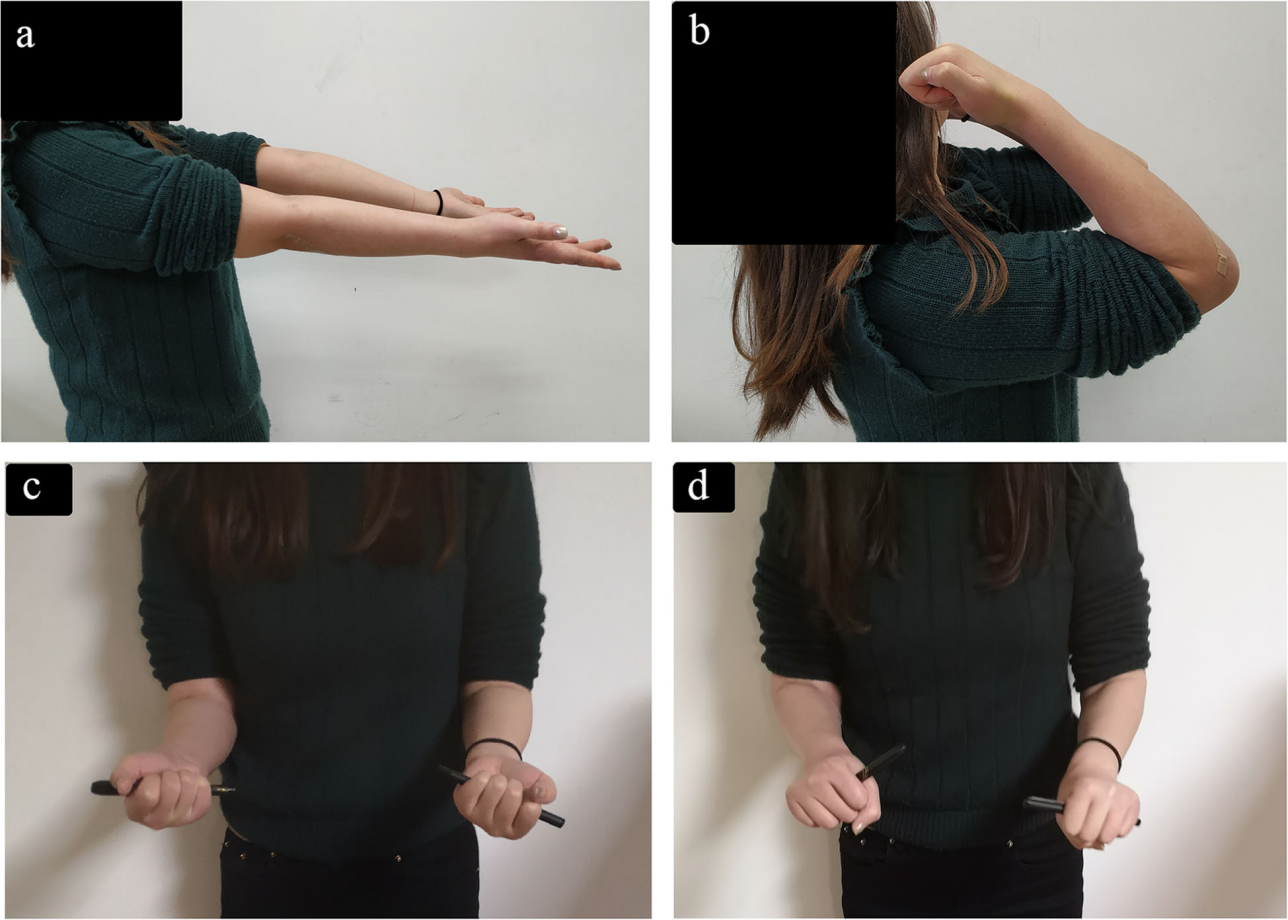
Functional follow-up at 6 months postoperatively. **a**: Normal range of elbow extension. **b**: Normal range of elbow flexion. **c**: Normal range of supination. **d**: Slight limitation of pronation

**Fig. 7:**
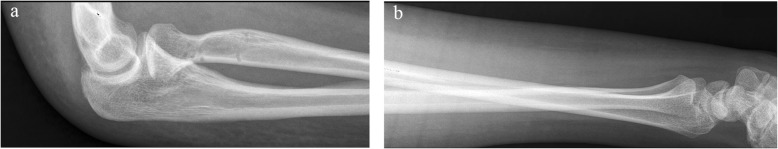
Radiographic follow-up at 6 months postoperatively. **a**: Lateral view of the elbow shows normal alignment between capitellum and the radial head. **b**: Lateral view of the wrist shows a reduced DRUJ

The long arm cast was kept for 4 weeks and replaced by an adjustable brace to allow a full range of flexion and extension. The K-wire was removed at 6 weeks postoperatively to allow a full range of forearm rotation. At 6 months postoperatively, the patient had regained a full range of flexion and extension, with normal supination and a slight limitation in pronation. Until 18 months postoperatively, the patient still had a slight limitation in pronation, with the Mayo elbow score being 85 and the Mayo wrist score being 90(Fig. 6). Radiographic findings revealed that the radial head and the DRUJ were in better positions, but there was still a criss-cross appearance between radius and ulna on the lateral view (Fig. 7).

## Discussion and conclusion

The injury of simultaneous dislocation of the radial head and the DRUJ without fracture was first introduced by Leung et al. [1] in 2002. They named this injury as a ‘Criss-Cross’ injury of the forearm, based on the ulna and radius forming a criss-cross appearance on the lateral radiograph. Until now, only 7 case reports were published about the ‘Criss-Cross’ injury. Among these case reports, 4 cases could be classified into severe types with complete dislocation of the radial head and the DRUJ [2–5], while the other 3 cases were less severe with subluxation of the radial head and DRUJ [1, 6, 7]. In our case, the patient also fell from the ground level and presented with a mild type of ‘Criss-Cross’ injury.

The pathologic changes of the ‘Criss-Cross’ injury were unclear in previous studies. The IOM of the forearm was believed to play the most important role in this injury [8]. Based on X-ray films and cadaveric studies, Leung et al. [1] hypothesized that the central band of the IOM might act as a pivot point between the radius and the ulna, producing paired rupture of the distal band and the proximal band. Unfortunately, the MRI did not cover the wrist so that the pathologic changes of the DRUJ and the distal IOM could not be confirmed.

The injury mechanism of ‘Criss-Cross’ injury was also ambiguous in previous studies. Bony contusion on the capitellum of the humerus and the proximal ulna indicated an axial and valgus force, while the proximal IOM being torn from the dorsal attachment of the radius indicated a pronated twisting force. According to a previous biomechanical study, the force across the central band of the IOM is nearly doubled with the forearm in neutral rotation as compared to supination or pronation with a constant axial load [9]. In other words, an axial load with the forearm in neutral rotation is more likely to disrupt the central band of IOM and create Essex-Lopresti injury. In contrast, a similar axial load with the forearm in hyperpronation might instead lead to rotation around the intact central band of the IOM and produce the paired dislocations in ‘Criss-Cross’ injuries. Normally, a radial head has a concave and smooth contour. However, in our case, CT scans and surgical findings revealed a convex and bumpy articular surface of the radial head. MRI images showed no obvious bony contusion or compressive fracture on the radial head, indicating that deformity might be an old injury. Similarly, Potter et al. [6] reported a chronic-appearing biconcave articular surface of the radial head with a midline ridge in their case. It was likely that the convex deformity of the radial head could disperse axial stress and transform it into transverse force, thus preventing radial head fracture and leading to radial head dislocation and rupture of the proximal IOM.

Simultaneous dislocation of the radial head and the DRUJ without fracture is a rare injury, and the pathologic changes and injury mechanism are unclear. This case demonstrates that the proximal IOM, especially the dorsal band, is injured in such injuries, while the central part of the IOM remains intact. This injury pattern distinguishes itself from Essex-Lopresti injury, which mainly involves rupture of the central band of the IOM.

## Supplementary information

**Additional file 1.**

**Additional file 2.**

## Data Availability

The datasets used during the current study are available from the corresponding author on reasonable request.

## Abbreviations

DRUJ: Distal Radio-Ulnar Joint
PRUJ: Proximal Radio-Ulnar Joint
IOM: Interosseous Membrane
CT: Computed Tomography
MRI: Magnetic Resonance Imaging
MCL: Medial Collateral Ligament
