# Supplementary figures and images for: Simultaneous dislocation of the radial head and distal radio-ulnar joint without fracture in an adult patient: a case report and review of literature
Source: BMC Surg. 2020 Apr 15;20:71. doi: 10.1186/s12893-020-00717-8 (PMC7160939; doi:10.1186/s12893-020-00717-8)

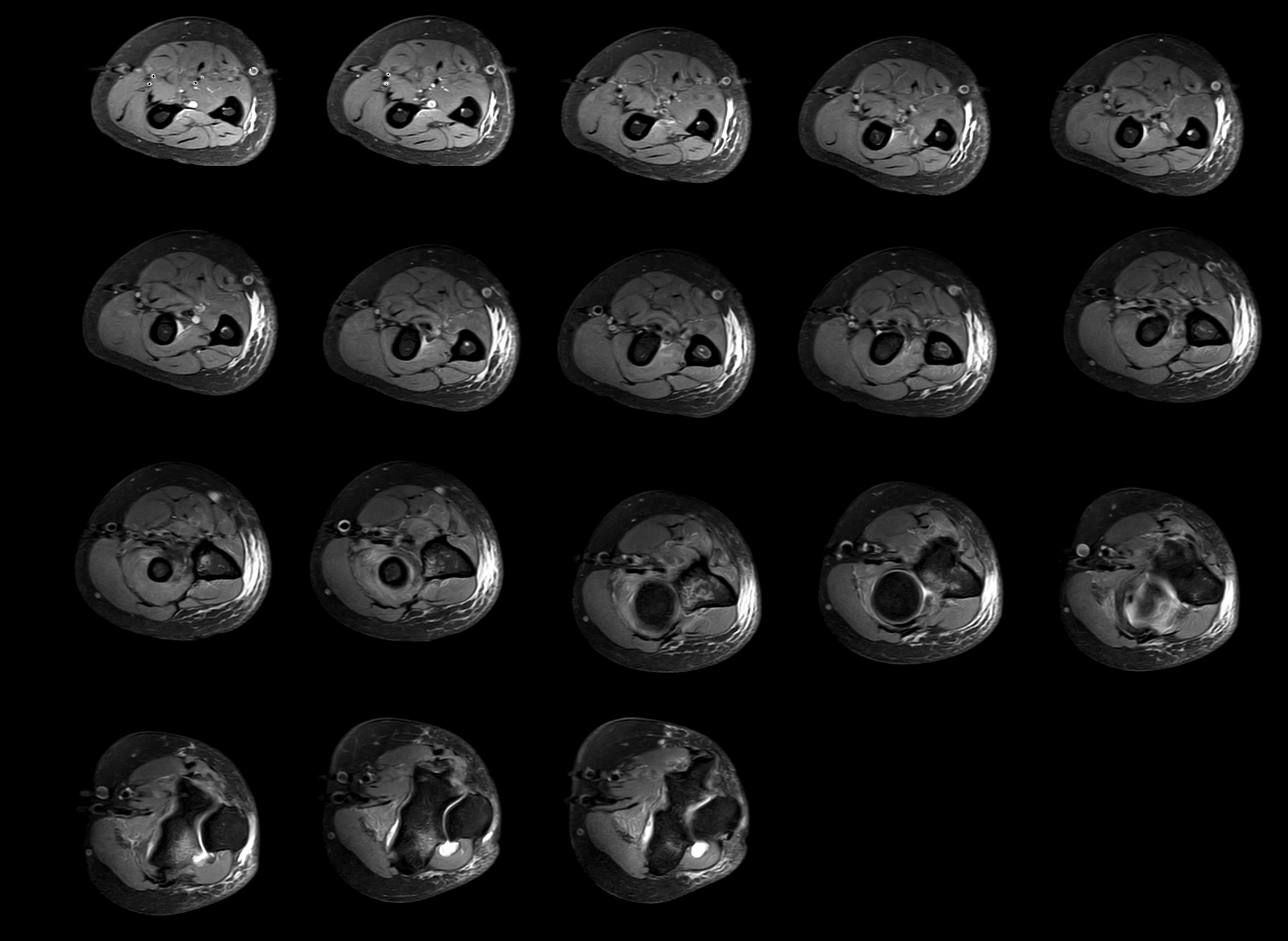

Supplement: Supplementary file 1 — Additional file 1. [file 12893_2020_717_MOESM1_ESM.tif]
